# Long-term outcomes of transaxillary versus video-assisted first rib resection for neurogenic thoracic outlet syndrome

**DOI:** 10.1093/icvts/ivac040

**Published:** 2022-03-09

**Authors:** Henrik Nuutinen, Jussi M Kärkkäinen, Mäkinen Kimmo, Aittola Voitto, Riekkinen Teemu, Saari Petri, Pesonen Janne

**Affiliations:** 1 Department of Surgery, Kuopio University Hospital, University of Eastern Finland, Kuopio, Finland; 2 Heart Center, Kuopio University Hospital, Kuopio, Finland; 3 Department of Radiology, Kuopio University Hospital, Kuopio, Finland; 4 Department of Rehabilitation Medicine, Kuopio University Hospital, Kuopio, Finland

**Keywords:** Neurogenic thoracic outlet syndrome, quality of life, video-assisted, transaxillary, long-term outcome, QuickDASH

## Abstract

**OBJECTIVES:**

This study compared the long-term outcomes in terms of clinical examinations and patient-reported outcome measures, between transaxillary and video-assisted thoracoscopic techniques for first rib resection in patients with neurogenic thoracic outlet syndrome.

**METHODS:**

The study population comprised patients who underwent first rib resection for neurogenic thoracic outlet syndrome at our institution between 2009 and 2016. All patients were recruited in a follow-up assessment in 2019, and those who agreed to participate were included in this study. Outcomes included examinations at the outpatient clinic and patient-reported outcome measures: Disabilities of Arm Shoulder and Hand Score and Cervical Brachial Symptom Questionnaire. The completeness of the rib resection was assessed on chest X-rays.

**RESULTS:**

:A total of 60 first rib resections (30 transaxillary + 30 video-assisted fully thoracoscopic approaches) were performed for neurogenic thoracic outlet syndrome in 47 patients between 2009 and 2016. Of these, 32 patients participated in the study including 18 who had transaxillary and 22 who had video-assisted thoracoscopic procedures. The mean follow-up was 5.9 (standard deviation: 2.2) years. The outcome was good or excellent after 15 (83.3%) and 17 (77.3%) procedures in the transaxillary and video-assisted thoracoscopic surgery groups, respectively (*P* = 0.709). There were no differences in patient-reported outcome measures between the 2 groups. Furthermore, the length of the residual first rib stump was similar in both groups.

**CONCLUSIONS:**

We found no differences in the long-term outcomes between the study groups. These results indicate that both transaxillary and purely thoracoscopic approaches offer favourable long-term outcomes following first rib resection in patients with neurogenic thoracic outlet syndrome.

**Figure:**
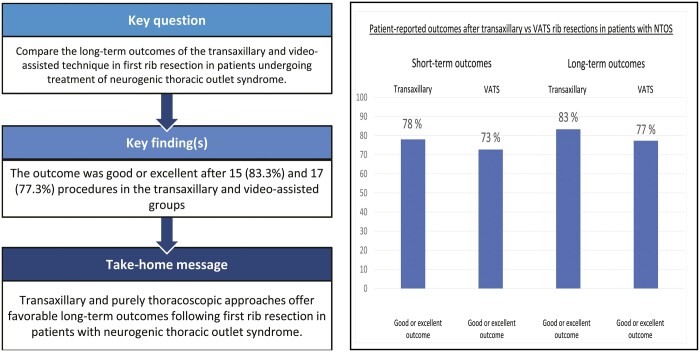

## INTRODUCTION

Neurogenic thoracic outlet syndrome (NTOS) is the most common manifestation of thoracic outlet syndrome [1]. Its symptoms are due to compression and irritation of the brachial plexus. Patients should meet 3 of 4 criteria for the diagnosis of NTOS according to the reporting standards of the Society for Vascular Surgery [2]. The 4 criteria are (i) local findings in the thoracic outlet, including pain and tenderness; (ii) peripheral findings due to nerve compression, including distal neurological changes, which are usually worse when the arms are placed overhead or dangling; (iii) absence of other probable diagnoses; and (iv) a positive response to scalene muscle injections. It is difficult to reach a definite diagnosis of NTOS, making it challenging to determine which patients are candidates for surgery.

The treatment of NTOS is primarily conservative, involving ergonomic changes to the patient’s work and daily life, together with physiotherapy and painkillers [2]. Surgery should be considered if the patient does not respond to conservative therapy. If surgery is selected, it involves first rib resection (FRR), which is often combined with scalenotomy [3]. The most common surgical techniques involve open transaxillary and supraclavicular approaches [3, 4]. However, with ongoing trends favouring minimally invasive surgery, a new resection technique based on video-assisted thoracoscopic surgery (VATS) has been introduced [5–7]. Although the thoracoscopic techniques described in the literature differ greatly, the technique used at our hospital is fully thoracoscopic. The VATS technique also provides better visibility of the surgical field, especially the posterior part of the rib compared to transaxillary FRR [8]. To date, only 1 study has published long-term results (> 2 years) of patients with NTOS operated on with a thoracoscopic technique: Hwang published long-term outcomes from a series of 8 patients, but only 1 patient had NTOS [9].

The goal of this study was to compare the long-term outcomes of the transaxillary technique and VATS in FRR in consecutive patients undergoing surgical treatment of NTOS. We evaluated the long-term outcomes in terms of patient-reported outcome measures (PROMs) and clinical examinations performed in outpatient clinics. We also measured the residual first rib stump to evaluate the correlation between stump length and any residual symptoms.

## PATIENTS AND METHODS

### Study design and ethics statement

This ambispective (i.e. data collected both retrospectively and prospectively from each patient) comparative cross-sectional cohort study was approved by the North-Savo institutional review board (ID : 342/2015). All patients provided written consent to participate in the study. All consecutive patients who underwent FRR for NTOS at a single academic teaching hospital between 2009 and 2016 were asked to participate in the study. The postoperative, short-term outcomes of these patients have been published All patients were invited to participate via a letter, and those willing to participate were included [5]in this follow-up study.

### Diagnostic criteria and workup

The diagnostic criteria for thoracic outlet syndrome (TOS) were pain and tenderness in the area of compression (scalene triangle, pectoral minor insertion site or both), evidence of nerve compression from distal symptoms and the absence of another potential cause. We did not perform scalene block testing. All patients underwent a diagnostic workup and received conservative physical rehabilitation for at least 6 months that was provided by a physiotherapist. Conservative therapy comprised manual physical therapy, therapeutic exercises and pain medication. Patients who failed to achieve satisfactory symptom resolution were referred to a senior vascular surgeon to be considered for surgery. Transaxillary FRR was performed using a standard approach originally described by Roos *et al*. [3]. VATS comprised complete FRR and division of the anterior and middle scalenus muscles via a fully thoracoscopic approach with a standard three-port technique. The transaxillary procedures were performed by a single senior vascular surgeon. The transition to the VATS approach occurred between 2012 and 2014, and the VATS procedures were performed by a single experienced thoracic surgeon. The operative techniques are described in detail in our prior publication [5].

### Data collection and outcome measures

We collected data retrospectively from the patients’ medical records, including diagnostic workup, index procedure and short-term outcomes at the 3-month follow-up. The clinical outcome at 3 months was graded as (i) no improvement or as (ii) partial, (iii) good or (iv) excellent recovery. Partial recovery was defined as some improvement with moderate residual symptoms. Good recovery was defined as minor residual symptoms. Excellent recovery was defined as the complete absence of symptoms. In the prospective part of the study, PROM questionnaires were sent with the invitation letter to evaluate long-term outcomes and patient satisfaction. The questionnaires included the shortened version of the Disabilities of Arm, Shoulder, and Hand (QuickDASH) Outcomes Measure and Cervical Brachial Symptom Questionnaire (CBSQ), both of which are recommended in the reporting standards of the Society for Vascular Surgery for TOS [2]. In addition, we used Beck’s Depression Inventory (BDI) and the Neck Disability Index (NDI) to identify possible factors that may confound the surgical outcomes. Patients also underwent a physical examination at the outpatient clinic, including the Adson, Spurling and Roos tests, and a thorough examination of the upper limb neurological status, including reflex, skin sensation and muscle strength tests. The patients also reported the TOS disability scale and pain scale scores [2]. The patient’s ability to work was estimated using questions covering a subjective assessment of their current ability to work (yes/no) and whether the procedure improved their working ability (yes/no). All clinical examinations were performed by the same senior physiatrist. During the examination, the patients were also interviewed regarding their reported outcomes. We used the same grading of symptom improvement as was used during the 3-month follow-up visit. A chest X-ray, limited to a clavicular projection, was taken to determine the potential posterior first rib remnant. The residual first rib length was measured by a senior radiologist.

### Statistical analysis

We used IBM SPSS Statistics, version 27 (IBM-SPSS Inc., Armonk, NY, USA) for all statistical analyses. Categorical variables are presented as the numbers and percentages of patients or procedures, as appropriate. Continuous variables are expressed as mean and standard deviation (SD). We used the Fisher exact test to compare categorical variables and the Mann–Whitney U test for nonparametric continuous variables. Results were considered statistically significant at *P*-values < 0.05.

## RESULTS

A total of 60 FRRs were performed for NTOS during the study period in 47 patients, of whom 13 underwent bilateral procedures. The first 30 underwent the transaxillary procedure and the latter 30 underwent VATS with a fully thoracoscopic approach. Of the 47 patients, 32 patients (68.1%) participated in the study, comprising 18 of 30 (60.0%) transaxillary procedures and 22 of 30 (73.3%) VATS procedures (*P* = 0.412). Both groups were comparable in terms of their general and clinical characteristics (Table 1). The mean age was 44.8 (SD: 12.5) years in the transaxillary group and 43.4 (SD: 10.8) years in the VATS group (*P* = 0.798). Most patients in both groups were female and had no other diseases.

**Table 1:** Preoperative characteristics of 32 patients with neurogenic thoracic outlet syndrome who underwent first rib resection (40 procedures)

Variable	Transaxillary procedure	Thoracoscopic procedure	*P*-value
Operations in the follow-up study	18	22	
Number of patients*	15	18	
Age, mean ± SD, years	44.8 ± 12.5	43.4 ± 10.8	0.798
Sex, female (%)	12 (66.7)	13 (59.1)	0.747
Operated arm: right (%)	9 (50.0)	11 (50.0)	1.000
Both sides operated (%)*	4 (22.2)	4 (18.2)	0.705
BMI, mean ±SD, kg/m^2^	27.3 ± 4.0	27.1 ± 3.9	0.778
Comorbidities			
Overweight (BMI > 30) (%)	7 (38.9)	5 (22.7)	0.315
Smoker (%)	3 (16.7)	2 (9.1)	0.745
Diabetes (%)	0 (0.0)	2 (9.1)	0.492
Hypertension (%)	3 (16.7)	8 (36.4)	0.286
Coronary artery disease (%)	1 (5.6)	1 (4.5)	1.000

Values are *n* (%) or mean ± SD.

* One patient underwent a transaxillary procedure and subsequent thoracoscopic surgery of the contralateral arm.

BMI: body mass index; SD: standard deviation.

### Long-term outcomes

The mean follow-up time was 70.2 (SD: 26.9) months overall, 94.5 (SD: 18.9) months in the transaxillary group and 50.4 (SD: 11.7) months in the VATS group (*P* < 0.001) (Table 2). At the long-term follow-up examination, the patients reported a normal working ability after 16 (88.9%) procedures in the transaxillary group and after 18 (81.8%) procedures in the VATS group (*P* = 0.673). The patients reported that their working ability had improved after surgery: 15/18 (83.3%) in the transaxillary group and 15/22 (68.2%) in the VATS group (*P* = 0.464). For most patients, the use of painkillers decreased after surgery compared with their preoperative situation. Two patients in both groups reported increased use of painkillers after surgery (Table 2).

**Table 2:** Long-term follow-up data for 40 first rib resections in patients with neurogenic thoracic outlet syndrome

Variable	Transaxillary procedure	Thoracoscopic procedure	*P*-value
Follow-up time, months	94.5 ± 18.9	50.4 ± 11.7	< 0.001
Normal working ability, number (%)	16 (88.9)	18 (81.8)	0.673
Working capacity improved with surgery, number (%)	15 (83.3)	15 (68.2)	0.464
Usage of painkiller decreased after surgery, number (%)	12 (66.7)	14 (63.6)	0.580
Usage of painkiller increased after surgery, number (%)	2 (11.1)	2 (9.1)	0.706
Queries			
Neck Disability Index, mean ± SD, score	11.6 ± 8.0	13.5 ± 9.9	0.619
Beck’s Depression Inventory, mean ± SD, score	6.4 ± 6.9	8.4 ± 10.8	0.737
Cervical Brachial Symptom Questionnaire, mean ± SD, score	43.0 ± 28.5	38.0 ± 24.6	0.819
Disabilities of Arm, Shoulder, and Hand Score (QuickDASH), mean ± SD, score	25.7 ± 16.5	33.6 ± 19.0	0.180
Would you go for surgery again?, mean ± SD, score 0-10, 0 = no, 10 = absolutely yes	7.8 ± 3.6	8.0 ± 3.7	0.657
Thoracic outlet syndrome disability scale, mean ± SD, score 0-10, 0= none, 10 = complete	2.6 ± 1.2	3.0 ± 2.0	0.527
Pain scale, mean ± SD, score 0-10, 0 = none, 10 = intolerable	3.3 ± 2.2	3.5 ± 2.9	0.925
Operated hand grip strength, mean ± SD, kg	33.6 ± 13.2	33.2 ± 9.2	0.946
Contralateral hand grip strength, mean ± SD, kg	33.8 ± 13.3	30.2 ± 10.0	0.527
Length of residual stump of operated first rib, mean ± SD, mm	30.9 ± 6.7	28.0 ± 9.3	0.286
Residual stump length more than 30 mm	9 (50.0)	9 (40.9)	0.750

Values are n (%) or mean ± SD.

SD: standard deviation.

The surgical outcome was considered good or excellent after 15/18 (83.3%) operations in the transaxillary group and after 17/22 (77.3%) operations in the VATS group (*P* = 0.709; Table 3) Total symptom relief was achieved after 1 transaxillary operation (5.6%) and after 7 VATS procedures (31.8%) (*P* = 0.054). In both groups, the proportion of procedures with good and excellent outcomes was greater at the long-term follow-up than at the short-term follow-up, with values of 83.3% versus 77.8%, respectively, in the transaxillary group and 77.3% versus 72.7%, respectively, in the VATS group (Table 3). The symptoms were unchanged after 3 procedures (16.7%) in the transaxillary group and after 2 procedures (9.1%) in the VATS group. Minor symptoms returned during the follow-up period after 12 (66.7%) procedures in the transaxillary group and after 12 (54.5%) procedures in the VATS group (*P* = 0.526). For 6 (50.0%) procedures in the transaxillary group and 8 (66.7%) in the VATS group, the symptoms returned more than 2 years after the initial procedure (*P* = 0.327; Table 3).

**Table 3:** Short-term and long-term outcomes of 40 first rib resections in patients with neurogenic thoracic outlet syndrome

Variable	Transaxillary procedure	Thoracoscopic procedure	*P*-value
Early follow-up status at approximately 3 months			
0. No significant improvement	0 (0.0)	2 (9.1)	
1. Partial recovery (residual symptoms)	4 (22.2)	4 (18.2)	
2. Good recovery (minor residual symptoms)	9 (50.0)	8 (36.4)	
3. Excellent recovery (fully asymptomatic)	5 (27.8)	8 (36.4)	0.737
Good or excellent recovery (2 + 3)	14 (77.8)	16 (72.7)	1.000
Late follow-up status, more than 50 months after surgery			
0. No significant improvement	3 (16.7)	2 (9.1)	
1. Partial recovery (residual symptoms)	0 (0.0)	3 (13.6)	
2. Good recovery (minor residual symptoms)	14 (77.8)	10 (45.5)	
3. Excellent recovery (fully asymptomatic)	1 (5.6)	7 (31.8)	0.054
Good or excellent recovery (2 + 3)	15 (83.3)	17 (77.3)	0.709
Whether the symptoms have come back over the years even a little?	12 (66.7)	12 (54.5)	0.526
Residual symptoms (n = 12 in both groups) have come back over 2 years after the operation	6 (50.9)	8 (66.7)	0.327
Reoperations	1 (5.6)	0 (0.0)	

Values are n (%).

### Patient-reported outcomes at the long-term follow-up

The mean NDI scores (maximum: 50) were 11.6 (SD: 8.0) in the transaxillary group versus 13.5 (SD: 9.9) in the VATS group (*P* = 0.619). The BDI scores (maximum: 63) were 6.4 (SD: 6.9) in the transaxillary group versus 8.4 (SD: 10.8) in the VATS group (*P* = 0.737). The mean CBSQ scores (maximum: 120) were 43.0 (SD: 28.5) in the transaxillary group versus 38.0 (SD: 24.6) in the VATS group (*P* = 0.819). The mean QuickDASH scores (maximum: 100) were 25.7 (SD: 16.5) in the transaxillary group versus 33.6 (SD: 19.0) in the VATS group, respectively (*P* = 0.180; Table 2). The mean TOS disability scale scores (maximum: 10) were 2.6 (SD: 1.2) in the transaxillary group versus 3.0 (SD: 2.0) in the VATS group, respectively (*P* = 0.527). The mean pain scale scores (maximum: 10) were 3.3 (SD : 2.2) in the transaxillary group versus 3.5 (SD: 2.9) in the VATS group (*P* = 0.925). The mean grip strengths were 33.6 (SD: 13.2) kg in the transaxillary group versus 33.3 (SD: 9.2) kg in the VATS group for the treated hand (*P* = 0.946) and were 33.8 (SD: 13.3) in the transaxillary group versus 30.2 (SD: 10.0) kg in the VATS group for the contralateral hand (*P* = 0.527). The patients were also asked whether they would undergo surgery again if they were given the choice (on scale of 0–10, where 0 = absolutely not and 10 = absolutely yes). The mean scores were 7.8 (SD: 3.6) in the transaxillary and 8.0 (SD: 3.7) in the VATS group (*P* = 0.657). The response score was 10 after 27 (out of 40) operations, and 6 responses were between 5 and 9. The response was between 0 to 2 after 7 operations, which is interpreted that these patients would not have undergone the surgery again.

### Residual stump and long-term complications

The length of the residual stump of the operated first rib was 30.9 (SD : 6.7) mm in the transaxillary group and 28.0 (SD: 9.3) mm in the VATS group and was not significantly different between the 2 groups (*P* = 0.286; Table 2). The stump length was >30 mm in 9 (50.0%) cases in the transaxillary group and in 9 (40.9%) cases in the VATS group (*P* = 0.750). The length of the residual stump over 30 mm had no correlation with the symptoms during the follow-up (Table 4). As short-term outcomes, we previously reported brachial plexus injury in 1 case [5]. The symptoms in this patient resolved completely with physiotherapy during the long-term follow-up. During the follow-up, 1 patient in the transaxillary group required reoperation because symptoms recurred several years after the index procedure. The patient underwent tenotomy of the pectoralis minor muscle as the secondary procedure (pectoralis minor compression syndrome). In this operation, the patient developed persistent iatrogenic Horner syndrome. One patient in the VATS group reported numbness of the operated chest.

**Table 4:** The first rib stump correlation with the long-term outcome for 40 first rib resections in patients with neurogenic thoracic outlet syndrome

Variable	Stump length > 30 mm (18 operations)	Stump length < 30 mm (22 operations)	*P*-value
Late follow-up status, more than 50 months after surgery			
0. No significant improvement	3 (16.7)	2 (9.1)	0.642
1. Partial recovery (residual symptoms)	1 (5.6)	2 (9.1)	
2. Good recovery (minor residual symptoms)	9 (50.0)	15 (68.2)	
3. Excellent recovery (fully asymptomatic)	5 (27.8)	3 (13.6)	0.430
Good or excellent recovery (2 + 3)	14 (77.8)	18 (81.8)	1.000

Values are n (%).

## DISCUSSION

Although there are plenty of publications on the treatment of NTOS, the literature on the long-term outcomes is scant. Moreover, there are no previous publications comparing long-term results and PROMs between the traditional transaxillary FRR and the fully thoracoscopic VATS approach. In this long-term follow-up study of patients who underwent surgical treatment of NTOS, we found no statistically significant outcome differences between the 2 approaches. After a mean follow-up of 4–8 years, 81.8%–88.9% of patients reported a normal working ability, and 68.2%–83.3% of the patients reported that surgery improved their ability to work. In addition, the use of painkillers decreased significantly after surgery in both groups. Furthermore, the PROMs indicated an objective improvement in quality of life.

The patients’ responses to whether they would undergo surgery again also demonstrated patient satisfaction at the long-term follow-up. This observation is supported by the low incidence of reoperation, which was necessary in only 1 patient. Furthermore, 77.3%–83.3% of the patients reported only minor or no residual symptoms at the long-term follow-up. This response is similar to the value in the study by Yin *et al.*, in which the success rate was 76% following transaxillary FRR for NTOS [10]. In the present study, 54.5%–66.7% of patients also reported that the symptoms returned to some extent during the follow-up period, as reported in previous studies [11, 12]. Approximately 20% of operated patients reported only partial recovery or no improvement after surgery. There can be several explanations for the “less than good” outcome in one-fifth of the patients. First is the diagnostic challenge of NTOS; it is possible that some patients who were operated on did not actually have NTOS because there is no definitive diagnostic test for this condition. Second is the recurrence of symptoms over time, perhaps, because of the formation of scar tissue that may accumulate around the brachial plexus. The third possibility is the incomplete decompression of the brachial plexus formation in the thoracic outlet. The fourth reason may be that some patients could have developed chronic pain that persisted even after decompression of the nerve. Altobelli *et al.* also stated that a good outcome of surgery generally means an improvement in symptoms, but not a total cure [11]. In the present study, most patients reported that their symptoms returned more than 2 years after surgery. This period is slightly longer that the 18 months reported in the study by Ambrad-Chalela *et al.* in 2004 [13]. The number of completely asymptomatic patients was numerically greater in the VATS group, although this did not reach statistical significance due to the small sample size. A shorter follow-up time may also contribute to this finding because symptoms often return over several years.

Using VATS, FRR can be performed under visual control. The first rib stump was shorter in the VATS group than in the transaxillary group, although the difference was not statistically significant. The first rib stump length was not correlated with the long-term outcomes. There is no consensus among experts regarding the length of the stump. Mingoli *et al.* previously reported that a stump greater than 3 cm is associated with residual symptoms [14]. In the present study, the stump was >3 cm in 40.9% of patients in the VATS group and in 50.0% of patients in the transaxillary group. In 1998, Molina *et al.* reported that subtotal removal of the first rib via a transaxillary approach may be acceptable to reduce the risk of neurogenic problems or vascular injury [15]. Video-assisted techniques may avoid this compromise because surgery is done under visual control. Therefore, the clinical significance of the stump length should be investigated in the future, especially in patients with NTOS.

PROMs are standardized, validated questionnaires and are therefore suitable for objective patient evaluation. We measured functional disability using QuickDASH. The mean score was slightly lower in the transaxillary group than in the VATS group, although the scores were similar to those in prior studies [16, 17]. By contrast, Gharagozloo *et al.* reported that the QuickDASH scores were significantly lower with robot-assisted techniques than in our study [18]. Symptoms were assessed using the CBSQ, and mean scores were lower in the VATS group than in the transaxillary group. These results were similar to those reported by Rochlin *et al.* in 2013 [19]. The NDI scores showed that the patients experienced a slight decrease in their ability to perform daily activities. The TOS disability scale indicated that our patients had mild disabilities. Using a pain scale, the patients reported occasional mild to moderate pain. The patients also completed the BDI to exclude other factors that may contribute to the symptoms. The results indicated that our patients were not depressed. Furthermore, we found no significant difference in the hand grip strength between the operated and contralateral sides.

Overall, 72.7%–77.8% of patients reported good or excellent results at the short-term follow-up, and 77.3%–83.3% of patients reported good or excellent results at the long-term follow-up. Thus, the present results suggest that the short-term outcome may predict the long-term outcome. Nevertheless, the symptoms returned during the long-term follow-up period, which was apparent as a decrease in the percentage of patients who reported an excellent result. Similarly, Altobelli *et al.* previously published that the outcomes of surgery deteriorate over time [11].

The retrospective nature of this study represents a limitation. In particular, the assessment of PROMs at the time of diagnosis would have been particularly helpful to gauge changes after surgery. Indeed, we might expect to see decreases in QuickDASH and CBSQ scores after surgery. The number of patients was relatively low. Although the participation rate was good, the reluctance of symptomatic patients to participate in the study might have caused selection bias. Selecting which patients are suitable for surgical treatment is particularly difficult for patients with NTOS. In addition, due to the study design, there was a statistically significant difference in the mean follow-up time between the 2 groups that may also cause bias in a small study.

The strength of the study was its multidisciplinary assessment using standardized, objective metrics at the long-term follow-up. Therefore, we believe that objective evaluations should be included in future studies involving patients with NTOS. The unique value of this study is the long follow-up of patients who underwent purely thoracoscopic techniques.

## CONCLUSIONS

Our results indicate that the outcomes of surgery did not differ between transaxillary and VATS for FRR in patients with NTOS, suggesting that both approaches provide favourable outcomes. The surgical techniques also resulted in satisfactory quality of life of patients in both groups. Surgeons should also be very judicious and critical when choosing on whom to operate.

## Abbreviations

BDI: Beck’s Depression Inventory
CBSQ: Cervical Brachial Symptom Questionnaire
FRR: first rib resection
NDI: Neck Disability Index
NTOS: neurogenic thoracic outlet syndrome
PROMs: patient-reported outcome measures
QuickDASH: shortened version of the Disability of the Arm, Shoulder, and Hand Outcome Measure
SD: standard deviation
TOS: thoracic outlet syndrome
